# Expression of CD25 fluctuates in the leukemia-initiating cell population of CD25-positive AML

**DOI:** 10.1371/journal.pone.0209295

**Published:** 2018-12-14

**Authors:** Yuki Kageyama, Hiroshi Miwa, Rino Arakawa, Isao Tawara, Kohshi Ohishi, Masahiro Masuya, Kazunori Nakase, Naoyuki Katayama

**Affiliations:** 1 Department of Hematology and Oncology, Mie University Graduate School of Medicine, Tsu, Mie, Japan; 2 School of Medicine, Mie University, Tsu, Mie, Japan; 3 Department of Transfusion Medicine and Cell Therapy, Mie University Hospital, Tsu, Mie, Japan; 4 Cancer Center, Mie University Hospital, Tsu, Mie, Japan; European Institute of Oncology, ITALY

## Abstract

CD25 is expressed on leukemic cells in 10–20% cases of acute myeloid leukemia (AML), and its expression is associated with poor prognosis. We reevaluated the relationship between CD25 expression and the leukemia-initiating cell (LIC) properties of AML using a patient-derived xenograft model. We divided lineage marker-negative (Lin^–^) CD34^+^CD38^–^ or Lin^–^CD34^+^ cells from CD25-positive AML into CD25-positive and -negative populations, and then transplanted each population into NOD.Cg-*Prkdc*^*scid*^*Il2rg*^*tm1Wjl*^/Sz mice. Leukemic engraftment was observed with both CD25-positive and -negative populations from three of nine CD25-positive AML patients. In two of those three patients, CD25-positive and -negative Lin^–^CD34^+^ cells engrafted at the primary transplantation led to leukemic engraftment at the secondary transplantation, in which engrafted cells contained both CD25-positive and -negative Lin^–^CD34^+^ AML cells. In an *in vitro* culture system, expression of CD25 was considerably induced in the CD25-negative population of Lin^–^CD34^+^ cells from two cases of CD25-positive AML. In one case, CD25-positive Lin^–^CD34^+^ cells gave rise to CD25-negative as well as -positive CD34^+^ cells. These observations suggest that there exist CD25-positive and -negative populations that can reconstitute CD25-positive AML in a patient-derived xenograft model, and that CD25 expression fluctuates in the LICs of AML.

## Introduction

A small but distinct population of leukemic stem cells (LSCs) initiates and propagates acute myeloid leukemia (AML) [1]. These LSCs are also thought to be the source of disease recurrence after the achievement of complete remission [2, 3]. The development of novel therapies targeting LSCs could improve the prognosis of AML [4]. In order to eradicate AML LSCs without harming normal HSCs, it is important to identify biological characteristics specific to LSCs. One type of assay used to detect LSCs is the patient-derived xenograft (PDX) model, which allows the identification of leukemia-initiating cells (LICs) [1, 5]. AML LICs have phenotypes and gene expression profiles similar to those of normal hematopoietic stem cells (HSCs) [1]. Several studies have described molecules, such as CD123 [6], CD47 [7], and TIM-3 [8], that are preferentially expressed on AML LICs.

CD25, also known as the α chain of interleukin-2 receptor, is strongly expressed on activated T cells and regulatory T cells. CD25 is aberrantly expressed on leukemic cells in a subset of AML, and its expression predicts adverse outcomes in those patients [9–14]. A recent study demonstrated that CD25-positive CD34^+^CD38^–^ AML cells develop AML when transplanted into immunodeficient mice, whereas CD25 is not expressed on normal HSCs [15]. However, it remains unclear whether CD25-negative CD34^+^CD38^–^ or CD25-negative CD34^+^ AML cells from CD25-positive AML patients have the capacity to engraft in immunodeficient mice. Here, we assessed the relationship between CD25 expression and LICs using a PDX model and analyzed the expression of CD25 on cultured CD25-positive and -negative CD34^+^ AML cells.

## Materials and methods

### Patient samples

All experiments were performed with authorization from the Independent Ethics Committee for Human Research at Mie University Graduate School of Medicine (protocol No. 1605). The study was conducted in accordance with the Declaration of Helsinki. Bone marrow (BM) and peripheral blood (PB) samples from AML patients were obtained and stored in Mie University Biobank Research Center. In this study, nine CD25-positive AML cases with detectable expression of CD34 were selected. Patient characteristics including age, gender, FAB classification, cytogenetics, internal tandem duplications in *FLT3* (*FLT3-ITD*), and white blood cell count are provided in Table 1. *FLT3-ITD* was analyzed using the TaKaRa PCR FLT3/ITD Mutation Set (Takara Bio, Kusatsu, Japan).

**Table 1:** Patient characteristics of CD25-positive AML.

Patient	Age (years)	Sex	FAB classification	Cytogenetics	*FLT3-ITD*	WBC (10^9^/l)
AML01	73	M	M1	46,XY,del(5)(q?)	WT	85.8
AML02	62	F	M6	46,XX	WT	61.6
AML03	55	F	M1	46,XX,t(1;2)(p36;p21)	WT	20.0
AML04	68	F	M1	45,XX,del(6)(p23),der(21;22)(q10;q11)	+	35.0
AML05	21	M	M1	47,XY,+8	+	58.8
AML06	55	M	M1	46,XY	+	140.0
AML07	28	M	M4	46,XX,t(6;9)(p23;q34)	+	10.4
AML08	67	M	M6	43,XY,del(5)(q13;q31),-7,add(13)(p11), der(16;21)(p10;q10),der(17;20)(q10;q10)	WT	23.0
AML09	63	M	M5b	45,X,-Y	+	22.4

AML, acute myeloid leukemia; F, female; *FLT3-ITD*, internal tandem duplications in *FLT3*; M, male; WBC, white blood cell count; WT, wild type; +, positive.

### Cell isolation

BM and PB mononuclear cells were isolated by density gradient centrifugation using Ficoll-Paque Plus (GE Healthcare Bio-Sciences, Uppsala, Sweden) within 48 hours of collection. Isolated cells were frozen in CellBanker 1 (Nippon Zenyaku Kogyo, Koriyama, Japan) in liquid nitrogen until use. To purify human leukemic cells from human/mouse chimeric BM of recipient mice, PE-conjugated anti–human CD45 (hCD45) antibody (BioLegend, San Diego, CA, USA) and anti-PE microbeads (Miltenyi Biotec, Bergisch Gladbach, Germany) were used.

### Flow cytometry and cell sorting

Isolated cells were stained with predetermined optimal concentrations of fluorophore-conjugated monoclonal antibodies. The following antibodies were used: anti-hCD3, hCD19, hCD25, hCD33, hCD34, hCD38, hCD45, and mouse CD45 (BioLegend). After discrimination of doublet and dead cells by staining with 7-aminoactinomycin D (7AAD, BioLegend), lineage (hCD3/hCD19) negative (Lin^–^) cells were analyzed and sorted on a BD FACSAria II (BD Biosciences, Franklin Lakes, NJ, USA). The fluorescence-minus-one controls were used to determine the gates for CD25, CD34, and CD38 positivity, and antibody isotype-matched controls were used for other antigens. The purity of sorted cells exceeded 95%. Data were analyzed using the FlowJo software v. 10.4.1 (Tree Star, Ashland, OR, USA). Data for phenotypic characterization of cultured cells were acquired on an LSRFortessa (BD Biosciences) and analyzed using FlowJo software v. 10.4.1.

### Cell culture

Cell cultures were performed in RPMI 1640 (Wako Pure Chemical, Osaka, Japan) supplemented with 10% fetal bovine serum (Sigma-Aldrich, St. Louis, MO, USA) at 37°C in a humidified 5% CO_2_ atmosphere. Sorted cells (3 × 10^5^ /ml) were incubated for 48 hours in 12-well plate (Nunc, Roskilde, Denmark) using medium with interleukin-3 (IL-3), granulocyte colony-stimulating factor (G-CSF), granulocyte-macrophage colony-stimulating factor (GM-CSF), erythropoietin (EPO), thrombopoietin (TPO), and stem cell factor (SCF). All of these cytokines were purchased from PeproTech (Rocky Hill, NJ, USA). Cytokines were used at the following concentrations: IL-3, 10 ng/ml; G-CSF, 10 ng/ml; GM-CSF, 10 ng/ml; EPO, 2 U/ml; TPO, 10 ng/ml; and SCF, 10 ng/ml.

### PDX assays

NOD.Cg-*Prkdc*^*scid*^*Il2rg*^*tm1Wjl*^/Sz (NSG) mice [16, 17] were purchased from The Jackson Laboratory (Sacramento, CA, USA) and used for PDX assays. Animal experiments were performed in accordance with institutional guidelines approved by Animal Research Committee, Mie University (protocol No. 28–6). For PDX assays, sorted cells were transplanted via the facial vein into NSG newborns within 48 hours of birth; the pups were irradiated with 120–150 cGy immediately before transplantation. Engraftment was defined as ≥0.1% human CD45^+^ cells. In addition, if the human CD45^+^ cells were CD3^–^ and CD33^+^ (≥90%), the engraftment was regarded as leukemic [18]. Mice were sacrificed by cervical dislocation after being anesthetized by inhalation of isoflurane.

## Results

### Both CD25-positive and -negative populations are detected in the CD34^+^CD38^–^ fraction of most cases of CD25-positive AML

**Fig 1:**
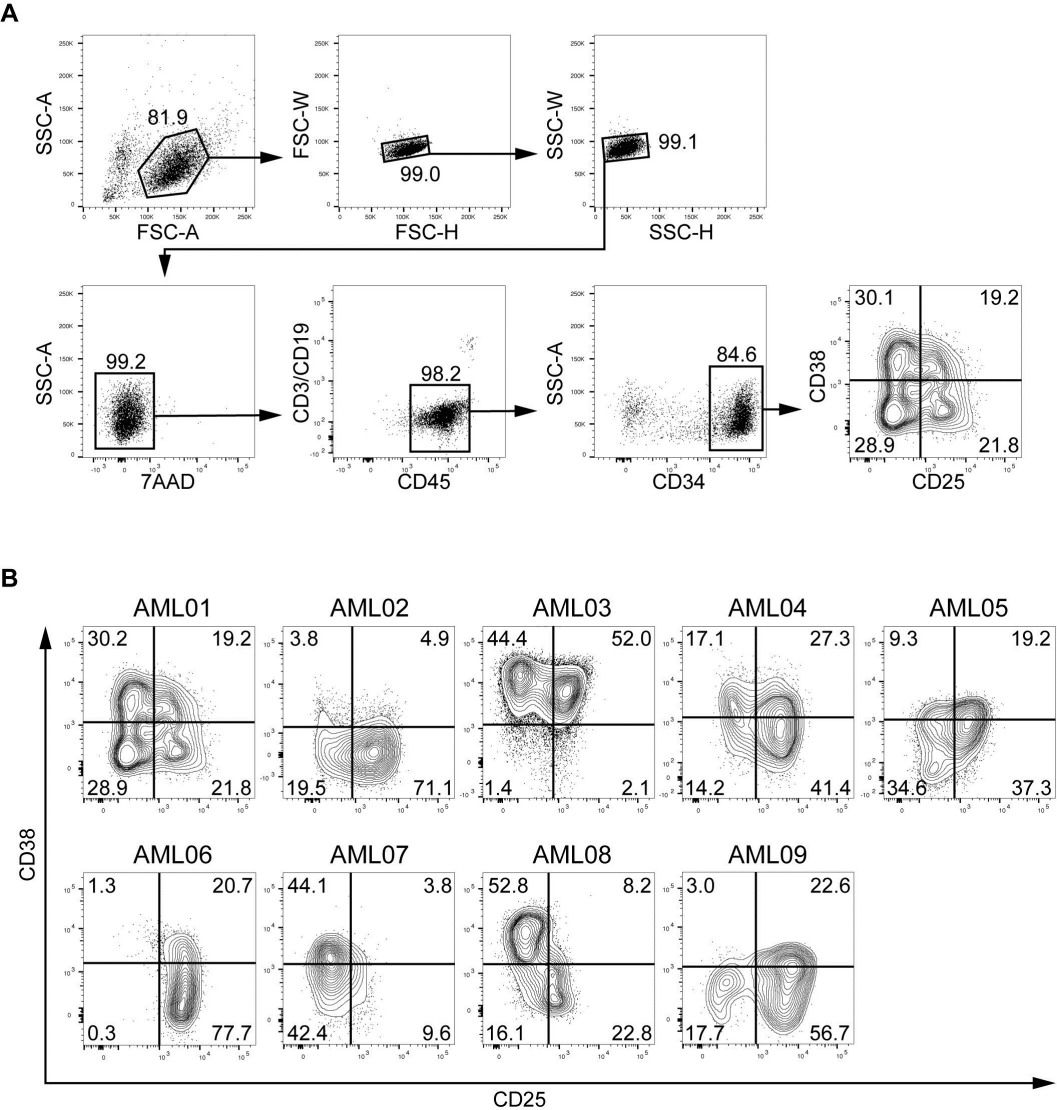
Gating strategy and flow-cytometric analysis of AML cells. A, Cells from AML01 were analyzed by flow cytometry. After discrimination of doublet and dead cells, the cells were gated as Lin^–^CD45^low^ cells, and then CD34^+^ cells were analyzed for CD38 and CD25 expression. B, CD38 and CD25 expressions on Lin^–^CD45^low^CD34^+^ population in nine AML patients are shown as contour plots. Percentages of each cell fraction are indicated in the plot areas.

We examined nine samples (one from each of nine cases) for expression of CD25 in the CD34^+^CD38^–^ fraction of AML cells. A representative gating strategy is shown in Fig 1A. In eight of the nine cases, both CD25-positive and -negative populations were detected in the CD34^+^CD38^–^ fraction. In the CD34^+^CD38^–^ fraction from AML03, the CD25-positive and -negative populations were small. In the remaining case (AML06), CD25 expression was detected on almost all CD34^+^CD38^–^ cells (Fig 1B).

### Both CD25-positive and -negative populations of CD34^+^CD38^–^ or CD34^+^ cells from CD25-positive AML undergo leukemic engraftment in a PDX model

**Fig 2:**
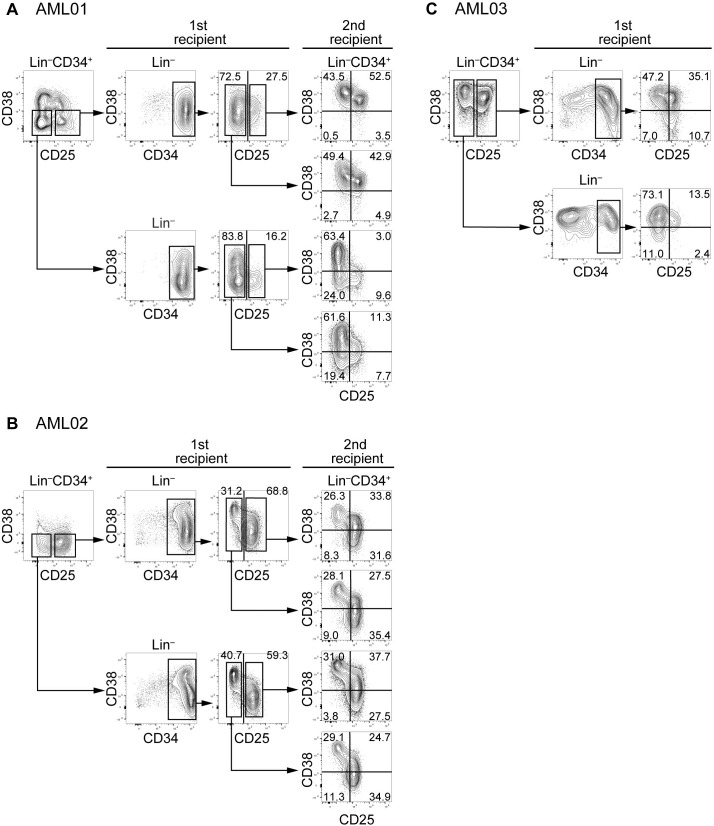
Reconstitution of human leukemia with distinct expression of CD25 at the primary and secondary transplantations in a PDX model. Lin^–^CD34^+^CD38^–^ cells from AML01 (A), 02 (B), and 03 (C) were sorted into CD25-positive and -negative populations, and then transplanted to primary recipients. Expression of CD25 on engrafted Lin^–^CD34^+^ cells was analyzed. Engrafted BM cells from AML01 and 02 were sorted again into CD25-positive and -negative populations, and then transplanted into secondary recipients. Expression of CD25 and CD38 on engrafted Lin^–^CD34^+^ cells in secondary recipients was analyzed.

We next investigated whether expression of CD25 was associated with LIC characteristics. For this purpose, we sorted CD25-positive and -negative populations of the CD34^+^CD38^–^ fraction from AML01, 02, and 06 or the CD34^+^ fraction from AML03, 04, 05, 07, 08, and 09 (the CD34^+^CD38^–^ fraction was very small in the latter five cases). Between 10,000 and 100,000 sorted cells were transplanted into irradiated newborn NSG mice via the facial vein. After leukemic engraftment was confirmed by checking human/mouse chimerism of PB at week 8 or 16, BM cells from mice sacrificed at week 18–20 were analyzed (Table 2). At the primary transplantation, the CD25-positive or -negative population was transplanted into two to seven mice. However, one to six of the mice transplanted with either CD25-positive or -negative cells from AML02, 03, 04, 05, 06, 07, or 09 died. Accordingly, only a few mice were available for analysis of leukemic engraftment. The engraftment rate was consistent with previous reports [3, 19, 20]. Leukemic engraftment was observed in the mice inoculated with the CD25-positive or -negative population from six cases (AML01–06), but not from the remaining three cases (AML07, 08, and 09). In three of the six engrafted cases (AML01, 02, and 03), leukemic engraftment was observed in both the CD25-positive and -negative populations (Fig 2).

**Table 2:** Results of PDX assays of CD25-positive AML.

		Primary transplantation						Secondary transplantation					
Patient	Sample type	Transplanted cells	No. of transplanted cells (× 10^3^)	No. of mice transplanted	No. of mice analyzed*	No. of mice engrafted	Type of engraftment	Transplanted cells	No. of transplanted cells (× 10^3^)	No. of mice transplanted	No. of mice analyzed*	No. of mice engrafted	Type of engraftment
AML01	PB	CD34^+^CD38^–^CD25^+^	90	2	2	2	Leukemic	CD34^+^CD25^+^	100	2	1	1	Leukemic
								CD34^+^CD25^–^	100	4	3	2	Leukemic
		CD34^+^CD38^–^CD25^–^	100	2	2	2	Leukemic	CD34^+^CD25^+^	20	2	2	2	Leukemic
								CD34^+^CD25^–^	20	4	3	2	Leukemic
AML02	PB	CD34^+^CD38^–^CD25^+^	100	3	2	2	Leukemic	CD34^+^CD25^+^	100	4	2	1	Leukemic
								CD34^+^CD25^–^	30	2	1	1	Leukemic
		CD34^+^CD38^–^CD25^–^	100	2	2	2	Leukemic	CD34^+^CD25^+^	100	3	2	1	Leukemic
								CD34^+^CD25^–^	60	2	1	1	Leukemic
AML03	BM	CD34^+^CD25^+^	100	4	3	3	Leukemic	–	–	–	–	–	–
		CD34^+^CD25^–^	100	4	2	2	Leukemic	–	–	–	–	–	–
AML04	BM	CD34^+^CD25^+^	33	7	1	1	Leukemic	hCD45^+^	100	4	3	0	–
		CD34^+^CD25^–^	33	2	1	1	Multi-lineage	–	–	–	–	–	–
AML05	PB	CD34^+^CD25^+^	100	4	2	0	No engraftment	–	–	–	–	–	–
		CD34^+^CD25^–^	100	4	2	2	Leukemic	hCD45^+^	10	3	3	0	–
AML06	PB	CD34^+^CD38^–^CD25^+^	50	4	3	1	Leukemic	hCD45^+^	30	3	3	0	–
AML07	BM	CD34^+^CD25^+^	10	2	1	0	No engraftment	–	–	–	–	–	–
		CD34^+^CD25^–^	10	4	1	0	No engraftment	–	–	–	–	–	–
AML08	BM	CD34^+^CD25^+^	100	3	3	0	No engraftment	–	–	–	–	–	–
		CD34^+^CD25^–^	100	3	3	0	No engraftment	–	–	–	–	–	–
AML09	BM	CD34^+^CD25^+^	15	3	2	0	No engraftment	–	–	–	–	–	–
		CD34^+^CD25^–^	15	2	2	0	No engraftment	–	–	–	–	–	–

–, data not available; AML, acute myeloid leukemia; BM, bone marrow; hCD45, human CD45; PB, peripheral blood.

* A number of mice transplanted with cells from AML03, 04, 05, 06, 07, or 09 died before the analysis.

### CD25-positive and -negative populations in engrafted leukemic cells were derived from either the CD25-positive or -negative population of CD25-positive AML

**Fig 3:**
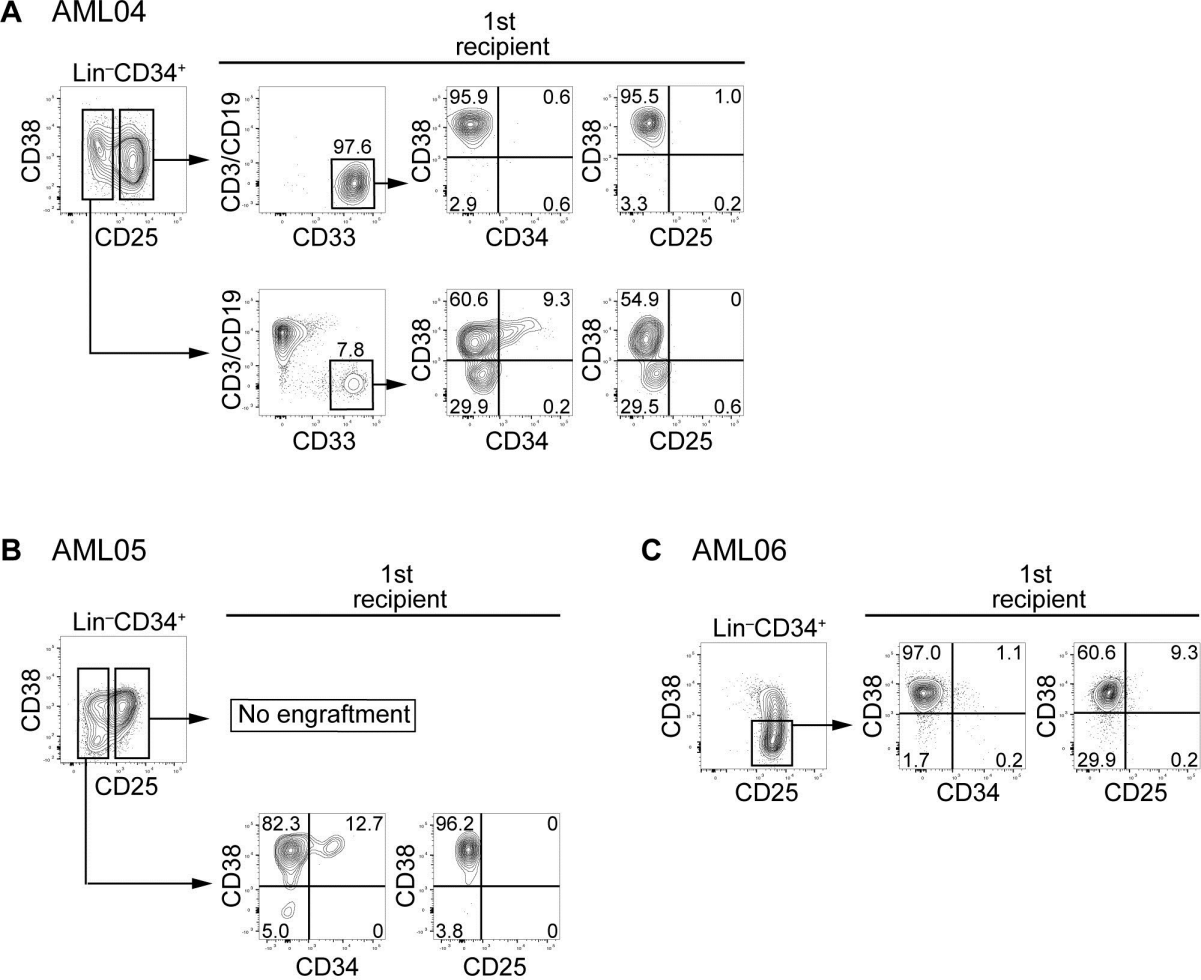
Reconstitution of human leukemia with distinct expression of CD25 at the primary transplantation into mice, with failure at the secondary transplantation. Lin^–^CD34^+^CD38^–^ cells from AML04 (A), 05 (B), and 06 (C) were sorted into CD25-positive and -negative populations, and then transplanted to primary recipients. Expression of CD3/CD19, CD33, CD34, CD38, and CD25 on engrafted cells was analyzed. The hCD45^+^ cells were sorted and transplanted to secondary recipients. No engraftment was observed in mice transplanted with hCD45^+^ cells from AML04, 05, or 06.

Phenotypic analyses of BM cells from mice with leukemic engraftment revealed that CD34 was expressed on the majority of engrafted cells from AML01 and 02 (Fig 2A and 2B). In AML03, a large number of engrafted cells from mice transplanted with the CD25-positive population expressed CD34, whereas CD34^+^ cells were found in approximately half of engrafted cells from mice transplanted with the CD25-negative population (Fig 2C). CD34 was negative or expressed at low levels on engrafted cells from the other three cases (AML04, 05, and 06) (Fig 3). When CD25-negative populations from AML01, 02, or 03 were transplanted, both CD25-positive and -negative Lin^–^CD34^+^ cells were found in engrafted cells at various proportions (Fig 2A, 2B and 2C).

The leukemic engrafted cells derived from AML04, 05, and 06 exhibited diminished expression of CD34 (Fig 3). In AML04, leukemic engraftment of CD25-positive Lin^–^CD34^+^ cells was observed, although CD25-negative Lin^–^CD34^+^ cells reconstituted multi-lineage hematopoiesis, including the T/B cell component (Fig 3A). The engrafted leukemic cells were negative for CD25 and CD34. CD25-positive Lin^–^CD34^+^ cells from AML05 did not engraft (Fig 3B). CD25-negative Lin^–^CD34^+^ cells recapitulated leukemia, but the engrafted leukemic cells did not express CD25. CD25-positive Lin^–^CD34^+^ cells alone were detected in AML06 (Fig 3C). These cells exhibited leukemic engraftment with CD25-negative CD34^–^ cells.

### CD25-positive and -negative populations in the engrafted leukemic cells at the primary transplantation reconstituted CD25-positive AML containing CD25-positive and -negative cells, or CD25-positive cells alone, at the secondary transplantation

Engrafted leukemic cells from five cases (AML01, 02, 04, 05, and 06) were subjected to secondary transplantation. At the secondary transplantation, three, four, and one mouse transplanted with cells from AML01, 02, and 04, respectively, died before the analysis. At the primary transplantation with CD25-positive or -negative population from AML01 and 02, the engrafted Lin^−^cells sustained CD34 expression (Fig 2A and 2B); therefore, Lin^–^CD34^+^ engrafted cells were again divided into CD25-positive and -negative populations. When these CD25-positive and -negative populations were transplanted into the secondary recipient mice, both CD25-positive and -negative populations established leukemia in which the expression of CD25 and CD38 in Lin^–^CD34^+^ cells varied. CD34^–^ leukemic cells alone were observed after the primary transplantation with the CD25-positive population from AML04 (Fig 3A). A substantial population of engrafted cells at the primary transplantation with CD25-negative Lin^–^CD34^+^ cells from AML05 or CD25-positive Lin^–^CD34^+^CD38^–^ cells from AML06 were negative for CD34 (Fig 3B and 3C). Accordingly, we isolated hCD45^+^ cells from the engrafted leukemic cells derived from AML04, AML05, and AML06 and subjected them to secondary transplantation. However, no engraftment was observed.

### Both CD25-positive and -negative cells in CD34^+^ cells of CD25-positive AML generate CD25-positive and -negative CD34^+^ cells in an *in vitro* culture system

**Fig 4:**
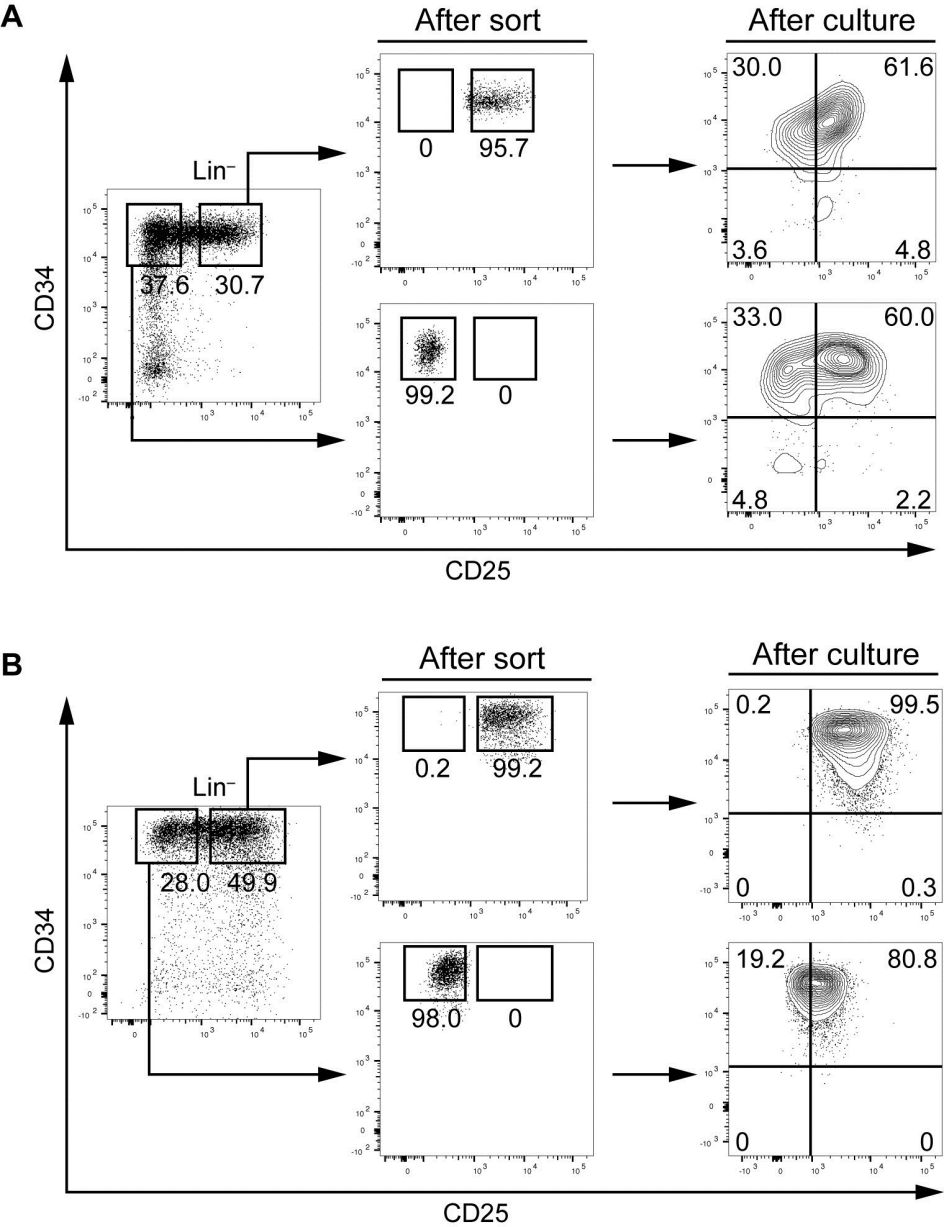
Cell culture of CD25-positive and -negative CD34^+^ cells from CD25-positive AML. CD25-positive and -negative CD34^+^ cells from AML01 and 05 were isolated and cultured for 48 hours at a concentration of 3 × 10^5^ /ml in 12-well plates in the presence of IL-3, G-CSF, GM-CSF, EPO, TPO, and SCF.

To determine whether CD34^+^ AML cells of CD25-positive AML alter expression of CD25, we cultured CD25-positive and -negative CD34^+^ cells from AML01 and 05 in the presence of cytokines. Forty-eight hours after the initiation of culture, cultured cells were harvested and analyzed for the expression of CD25 and CD34. Expression of CD25 was induced in a considerable fraction of the cultured cells derived from CD25-negative cells from AML01 and 05, whereas CD25-positive cells retained expression of CD25. CD25-positive cells from AML01 yielded a detectable population of CD25-negative CD34^+^ cells (Fig 4).

## Discussion

Recently, stemness genes expressed in AML cells were reported to be associated with increased engraftment potential in immunodeficient mice as well as unfavorable clinical outcome [18, 21, 22]. The gene expression signature of CD25-positive AML is significantly enriched in these stemness genes [11]. Saito *et al*. reported that CD25 is highly expressed on LSCs, but not on normal HSCs, suggesting that it may be an LSC-specific marker [15]. The purpose of this study was to determine the relationship between CD25 expression and LIC properties of CD25-positive AML. It is generally accepted that LICs are CD34^+^CD38^–^, although the improvement of PDX models revealed that LICs also exist in the CD34^+^CD38^+^ fraction [23] and even in the CD34^–^ fraction [24]. Therefore, we investigated the association of CD25 expression with LIC characteristics in the CD34^+^CD38^–^ population, when such cells were detectable, or otherwise in the CD34^+^ population. LICs are defined by their ability to initiate leukemia in a recipient mouse and grow out after re-transplantation into secondary recipients (and preferably in tertiary recipients) [1, 25]. Our results indicated that LICs from CD25-positive AML that fulfill this definition are present not only in the CD25-positive population but also in the CD25-negative population. In addition, CD25-positive leukemia developed following transplantation of a CD25-negative population, and *vice versa*. Our data obtained in culture also support the notion that CD25-negative and -positive CD34^+^ cells from CD25-positive AML can become CD25-positive and -negative, respectively, while retaining expression of CD34. On the basis of these data, we assumed that the expression of CD25 fluctuates in AML cells with phenotypic features of LICs.

‘LSC markers’ should satisfy the following conditions: (1) low or undetectable expression in normal tissues, especially normal HSCs, and (2) universal expression in LSCs. Because previous studies reported negative or relatively low expression of CD25 on normal HSCs [15], CD25 meets condition (1). On the other hand, our observation in AML01, 02, and 03 that both the CD25-positive and -negative populations had leukemia-initiating capacity at the primary or secondary transplantation indicated that CD25 does not satisfy condition (2).

Recent research showed that the LICs of human chronic myeloid leukemia (CML) are composed of CD25-positive and -negative populations, and that IL-2 may be involved in the generation and maintenance of CML LICs, suggesting that targeting IL-2/CD25 axis could be effective for eradicating CML LSCs [26]. Our results showed that the CD25-negative population of Lin^–^CD34^+^CD38^–^ or Lin^–^CD34^+^ cells from CD25-positive AML patients reconstituted leukemia at the primary or secondary transplantation in the absence of the CD25-positive population. Therefore, it is plausible that the cells with LIC phenotype from CD25-positive AML can generate and maintain leukemia irrespective of their expression of CD25.

Gönen *et al*. reported a high frequency of *FLT3-ITD* in CD25-positive AML [11]. In this study we detected *FLT3-ITD* in five of nine patients with CD25-positive AML. Leukemic cells of AML01 and 02, which exhibited leukemic engraftment at the primary and secondary transplantations, did not harbor *FLT3-ITD*, whereas *FLT3-ITD* positivity was observed in cells from AML07 and 09 that did not develop leukemic engraftment. With regard to CD25-positive AML, it is possible that leukemic engraftment potential in a PDX model or CD25 expression profile on LICs of CD25-positive AML is not associated with *FLT3-ITD*. To address this issue, further studies using a large number of CD25-positive AML cases are required.

Taken together, our data demonstrate that LICs of CD25-positive AML exist in both CD25-positive and -negative populations, and that CD25 expression can fluctuate in LICs of CD25-positive AML. A detailed analysis of different subsets of LICs would provide useful information that could facilitate development of LSC-targeting therapy.
